# Supplementary material for: Primary Tumor Resection in Synchronous Metastatic Colorectal Cancer Patients Treated with Upfront Chemotherapy plus Bevacizumab: A Pooled Analysis of TRIBE and TRIBE2 Studies
Source: Cancers (Basel). 2023 Nov 16;15(22):5451. doi: 10.3390/cancers15225451 (PMC10670103; doi:10.3390/cancers15225451)
Supplement: Supplementary file 1 [file cancers-15-05451-s001.zip › cancers-2677142-supplementary.pdf]

Table S1. Uni- and multivariate analyses for progression-free survival.

CHARACTERISTICS	N		UNIVARIATE ANALYSIS		MULTIVARIATE ANALYSIS	
	999	Months	HR (95% CI)	*p*	HR (95% CI)	*P*
**Treatment Arm**						
Doublet CT + bevacizumab	502	9.6	1	***<0.001***	1	***0.001***
FOLFOXIRI +bevacizumab	497	11.9	0.74 (0.65-0.84)		0.76 (0.66-0.87)	
**Age**						
≥70 years	141	10.8	1	*0.70*	-	
<70 years	858	10.6	0.96 (0.80-1.16)			
**ECOG PS**						
1-2	131	7.8	1	***<0.001***	1	***<0.001***
0	868	11.2	0.57 (0.47-0.68)		0.61 (0.50-0.75)	
**Liver-Only Disease**						
Yes	276	12.2	1	***0.0006***	1	***0.018***
No	721	9.9	1.29 (1.11-1.49)		0.69 (0.51-0.94)	
Missing	2	24.3				
**Number of metastatic sites**						
1	352	12.3	1	***<0.001***	1	***<0.001***
>1	645	9.7	1.44 (1.26-1.66)		1.80 (1.34-2.40)	
Missing	2	24.3				
**Resected Primary Tumor**						
No	486	10.0	1	***0.0005***	1	***0.032***
Yes	513	11.1	0.80 (0.70-0.91)		0.85 (0.74-0.99)	
**Gender**						
Female	414	10.9	1	*0.20*	-	
Male	585	10.6	1.09 (0.96-1.24)			
**Site of Primary Tumor**						
Right	360	10.0	1	*0.17*	-	
Left	617	11.2	0.91 (0.79-1.04)			
Missing	22	9.4				
***RAS/BRAF* Mutational Status**						
RAS/BRAF wild-type	193	12.3	1		1	
RAS mutated	576	10.3	1.24 (1.04-1.47)	***0.0154***	1.24 (1.04-1.47)	***0.016***
BRAF mutated	84	7.4	1.82 (1.40-2.38)	***<0.001***	1.93 (1.47-2.54)	***<0.001***
Missing	146	11.6				

N: number; HR: Hazard Ratio; CI: Confidence Interval; ECOG PS: Eastern Cooperative Oncology Group Performance Status.

Table S2. Uni- and multivariate analyses for overall survival.

CHARACTERISTICS	N		UNIVARIATE ANALYSIS		MULTIVARIATE ANALYSIS	
	999	Months	HR (95% CI)	*p*	HR (95% CI)	*P*
**Treatment Arm**						
Doublet CT + bevacizumab	502	23.0	1	***0.016***	1	*0.13*
FOLFOXIRI +bevacizumab	497	26.9	0.84 (0.72-0.97)		0.88 (0.75-1.04)	
**Age**						
≥70 years	141	23.6	1	*0.23*	-	
<70 years	858	24.8	0.88 (0.72-1.08)			
**ECOG PS**						
1-2	131	15.2	1	***<0.001***	1	***<0.001***
0	868	26.3	0.46 (0.37-0.56)		0.49 (0.39-0.60)	
**Liver-Only Disease**						
Yes	276	31.4	1	***<0.001***	1	*0.30*
No	721	22.7	1.48 (1.25-1.75)		0.83 (0.58-1.18)	
Missing	2	28.4				
**Number of metastatic sites**						
1	352	30.3	1	***<0.001***	1	***0.0061***
>1	645	21.6	1.55 (1.32-1.81)		1.59 (1.14-2.22)	
Missing	2	28.4				
**Resected Primary Tumor**						
No	486	22.5	1	***<0.001***	1	***0.018***
Yes	513	26.6	0.78 (0.67-0.90)		0.82 (0.70-0.97)	
**Gender**						
Female	414	26.1	1	*0.52*	-	
Male	585	24.2	1.05 (0.91-1.22)			
**Site of Primary Tumor**						
Right	360	20.6	1	***<0.001***	1	***0.018***
Left	617	26.9	0.72 (0.62-0.84)		0.81 (0.68-0.97)	
Missing	22	21.8				
***RAS/BRAF* Mutational Status**						
RAS/BRAF wild-type	193	35.8	1			
RAS mutated	576	23.7	1.73 (1.41-2.14)	***<0.001***	1.69 (1.36-2.09)	***<0.001***
BRAF mutated	84	14.0	3.17 (2.36-4.27)	***<0.001***	3.03 (2.20-4.16)	***<0.001***
Missing	146	26.5				

N: number; HR: Hazard Ratio; CI: Confidence Interval; ECOG PS: Eastern Cooperative Oncology Group Performance Status.
